# Ultrastructural changes in methicillin-resistant *Staphylococcus aureus* (MRSA) induced by metabolites of thermophilous fungi *Acrophialophora levis*

**DOI:** 10.1371/journal.pone.0258607

**Published:** 2021-10-14

**Authors:** Shivankar Agrawal, Jusna Nandeibam, Indira Sarangthem

**Affiliations:** 1 Indian Council of Medical Research (ICMR), Delhi, India; 2 Department of Microbiology, Institute of Bioresources and Sustainable Development, A National Institute of Department of Biotechnology, Government of India, Imphal, Manipur, India; Bangabandhu Sheikh Mujibur Rahman Agricultural University, BANGLADESH

## Abstract

*Staphylococcus aureus* and Methicillin-resistant *S*. *aureus* (MRSA) remains one of the major concerns of healthcare associated and community-onset infections worldwide. The number of cases of treatment failure for infections associated with resistant bacteria is on the rise, due to the decreasing efficacy of current antibiotics. Notably, *Acrophialophora levis*, a thermophilous fungus species, showed antibacterial activity, namely against *S*. *aureus* and clinical MRSA strains. The ethyl acetate extract of culture filtrate was found to display significant activity against *S*. *aureus* and MRSA with a minimum inhibitory concentration (MIC) of 1 μg/mL and 4 μg/mL, respectively. Scanning electron micrographs demonstrated drastic changes in the cellular architecture of metabolite treated cells of *S*. *aureus* and an MRSA clinical isolate. Cell wall disruption, membrane lysis and probable leakage of cytoplasmic are hallmarks of the antibacterial effect of fungal metabolites against MRSA. The ethyl acetate extract also showed strong antioxidant activity using two different complementary free radicals scavenging methods, DPPH and ABTS with efficiency of 55% and 47% at 1 mg/mL, respectively. The total phenolic and flavonoid content was found to be 50 mg/GAE and 20 mg/CAE, respectively. More than ten metabolites from different classes were identified: phenolic acids, phenylpropanoids, sesquiterpenes, tannins, lignans and flavonoids. In conclusion, the significant antibacterial activity renders this fungal strain as a bioresource for natural compounds an interesting alternative against resistant bacteria.

## 1. Introduction

*Staphylococcus aureus* is a commensal and opportunistic pathogen known to cause numerous mild to life-threatening infections in both human and animals. *S*. *aureus* remains one of the leading causative agents of morbidity and mortality worldwide [1]. This is to a large extent due to antibiotic-resistant strains, in particular methicillin-resistant *S*. *aureus* (MRSA) [2]. Currently, there is a pressing need to discover novel and effective antibacterial drugs to counteract this dramatic emergence of multi drug resistant (MDR) infections. Traditional healers have been using various natural resources for improving human health with comparatively less or no side effects. Globally, numerous medicinal plants have been explored worldwide to find novel drug molecules to fight the risk of ever increasing human diseases [3]. However, large scale harvesting of medicinal plants has already become a major threat to biodiversity [4]. As an alternative, the microbes, which live in diverse habitats, may offer tremendous potential sources of therapeutic compounds. The microbial drug era began after the discovery of penicillin from fungi *Penicillium notatum* by Alexander Fleming. At the present scenario, microorganisms have played a phenomenal contribution in the healthcare sector globally [5]. There is an immense evidence available in the literature which indicates that microorganisms are capable of producing numerous bioactive molecules for drug discovery which plays an important role in the pharmaceutical industry [6]. However, microbes from an unexploited environment are very less to known [7]. Therefore, researchers have curved their interest into extremophiles [8], thermophiles [9], radiation-resistant extremophiles [10], and acid mine waste extremophiles [11]. The present study was aimed to unveil the potential of a thermophilous *Acrophialophora* species IBSD-19 from the natural hot spring of Northeastern India for its antibacterial activity against *S*. *aureus* isolates. Identification of the fungi was done based on morphological properties and analysis of 18S rRNA sequencing. In addition, scanning electron microscope (SEM) observations were carried out to probe the possible antibacterial mechanism against *S*. *aureus* and MRSA.

## 2. Material and methods

### 2.1 Collection of samples

The sampling was carried out at Resu belpara popularly known as Resu, is the headquarters of North Garo Hills District in the state of Meghalaya in India. Hot spring, Bakra is situated in the North Garo Hills District of Meghalaya, India. This hot spring is located between 25°54’ N latitude and 90°36’ E longitude at an altitude of 3667ft. Several water and sediment samples were collected at different areas within the hot spring in the month of September 2013. After collection, the samples were brought to the laboratory for isolation of thermophilous fungi. The water and sediment samples are subjected to pretreatment at a higher temperature of 55 °C for 2–7 days to enhance the population of thermophilous fungi.

### 2.2 Isolation and preservation of pure fungal strain

The samples were diluted at 10 to 100-fold in sterile water and samples were used directly as inoculum. A volume of 100 μL of each dilution was spread onto potato dextrose agar (PDA, Hi Media, Mumbai, India) plates (39 gm PDA in 1,000 mL of distilled water, supplemented with antibacterial agents; 100 mg/L streptomycin and 50 mg/L penicillin). The inoculated plates were incubated at 45 ± 2 °C for 21 days. After 2 days of incubation the plates were examined daily for the presence of fungal growth. Distinct fungal colonies were then transferred to fresh media plates and allowed to grow for 5–7 days at 45 °C. After microscopic examination of the culture at 40× magnification using light microscope, agar plugs containing the pure fungus were placed in sterile distilled water and stored at 4 °C. Fungal viability was determined periodically as per the protocol described by Kjer et al. [12].

### 2.3 Fermentation and extraction of metabolites

Extraction of fungal secondary metabolites was performed according to the method described by Agrawal et al. [13] with slight modification. The pure culture of 10-day old fungal isolate was inoculated into 500 mL Erlenmeyer flasks containing 200 mL of potato dextrose broth (PDB, Hi Media) and incubated at 45 ± 2 °C for 15 days under shaking conditions at 150 rpm. Afterwards, the fermented broth was filtered to separate the mycelia and the filtrate. The fermentation broth (culture filtrate) was sequentially extracted twice with each hexane, chloroform, and ethyl acetate at room temperature under fume hood and obtained organic extracts were further concentrated in a Rotary evaporator (BUCHI Rota vapor R-100). The residue obtained was stored in glass vials in the freezer until further use.

### 2.4 Morphological identification of the fungal strain

Fungal isolate was identified initially by morphological characterization and microscopic observation using lactophenol cotton blue stain. The slides were examined under the light microscope (Labovision, India). The isolate was identified up to generic level using various fungal monographs [14] and references from the online database MycoBank (http://www.mycobank.com). Subsequently, the isolate was identified through molecular characterization by the analyses of nuclear ribosomal internal transcribed spacer (ITS) regions.

### 2.5 Molecular characterization of fungal strain

Fungal genomic DNA was extracted from the 7 days old fungal culture grown in potato dextrose broth (PDB) by using minor modification as described by Hermosa et al. and Kamala et al. [15, 16]. The ITS region of the fungi was amplified using the primer pairs ITS1/ITS4 (5′-TCC GTA GGT GAA CCT GCG G-3′/ 5′-TCC TCC GCT TAT TGA TAT GC-3′) and ITS1F/ITS4B (5′CTTGGTCATTTAGAGGAAGTAA-3′/ 5′-CAGGAGACTTGTACACGGTCCAG-3′) as described by White et al. [17] in an automated thermocycler (Bio-rad-C1000 Thermal Cycler). The PCR reaction mix of 25 μL reaction volumes consist of 2.5 μL of 10x buffer, 1.5 μL of MgCl_2_ (25 mM), 0.5 μL of dNTP (10 mM), 1 μL of ITS1 primer (5 pm), 1 μL of ITS4 primer (5 pm), 0.2 μL of Taq Polymerase (2.5 U), 3 μL of DNA sample (5 μg/ mL) and 15.3 μL sterile Milli Q Water. The PCR reaction was carried out in C1000 Touch^™^ Thermal Cycler (Bio-Rad, U.S.A) with conditions as follows: denaturation for 5 min at 94 °C, 33 elongation cycles (50 s at 95 °C, 50 s at 56 °C, 1 min at 72 °C) final extension for 10 min at 72 °C and last at 4 °C. Negative controls were used to confirm the absence of contamination. The final products were analyzed on 1% Agarose (Sigma-Aldrich, St. Louis, USA) and visualized under UV light using Gel imaging system (BioRad, Chemi Doc, MP). The sequences of the PCR products were analyzed by Finch TV 1.4.0 [18]. The sequences were then compared with the NCBI GenBank database by the BLASTN program. Phylogenetic relationships were estimated using MEGA 6 [19].

### 2.6 Bacterial strain and culture conditions

*Staphylococcus aureus* (ATCC 11632) was purchased from the American Type Culture Collection (Manassas, VA, USA) and MRSA was obtained from the Regional Institute of Medical Sciences (RIMS), Imphal, India. Safety precautions were undertaken before handling the pathogen. The pathogenic strain was carefully transferred to the laboratory and Biosafety level 2 (BSL 2). Microbial stock cultures were stored in cryoinstant vials containing 20% (v/v) glycerol at -80 °C.

A loopful of pure colonies of the test microorganisms were inoculated into 10 mL nutrient broth and incubated at 37 °C for 4 h. The turbidity of the actively growing bacterial suspension was adjusted to match the turbidity standard of 0.5 McFarland standard (1.5 × 10^8^ CFU/ mL at 600 nm), prepared by mixing 0.5 mL of 1.75% (w/v) barium chloride dihydrate to 99.5 mL of 0.18 M (v/v) sulphuric acid with continuous mixing. The bacterial suspension so prepared was used for testing their sensitivity to the samples under investigation [13].

### 2.7 Antibacterial activity

#### 2.7.1 Preliminary antimicrobial activity

Agar well-diffusion method described by Agrawal et al. [20] was used to determine the antibacterial activity. Mueller Hinton agar no. 2 (Hi Media, India) was prepared and cooled to 40–45 ºC and bacterial inoculum (1.5×10^8^ CFU/mL, 0.5 McFarland) prepared above was then added aseptically to the molten agar and poured into sterile petri dishes to give a solid plate. Wells (6 mm diameter) were made in each of these plates using sterile cork borer. The wells were filled with 10 μL of test fraction (1 mg/mL of methanol). The plates were first incubated at 4 ºC for 15 min and then at 37 ºC for overnight. The antibacterial potential of each fraction; hexane, chloroform, and ethyl acetate were determined for *S*. *aureus* and MRSA in terms of zone sizes around each well. The diameters of zones of inhibition (ZOI) produced by the test sample were recorded and compared with control antibiotics; streptomycin, ampicillin and chloramphenicol (1 mg/mL, Sigma-Aldrich). For control, pure solvent (methanol) was used. The experiment was performed in triplicates to minimize the error.

#### 2.7.2 Minimum inhibitory concentration

The MIC of active fraction was determined by a microdilution assay in 96-well microtiter plates according to the dilution assay in line with the Clinical and Laboratory Standards Institute (CLSI) recommendations [21]. The fresh cultures were prepared at 24 h broth cultures of *S*. *aureus* and MRSA and OD was set at 0.5 McFarland standard (1.5 × 10^8^ CFU/mL) at 600 nm with respective media broth. The bacterial suspension and the serially diluted active ethyl acetate fraction was added to 96-well plates to obtain the final volume of 200 mL. The microplates were incubated at 37 °C with continuous shaking. After 18 h, the optical density at 600 nm was measured with a microplate reader. Streptomycin was used as positive controls. The MIC values were determined at the zero-optical density concentration. All experiments were repeated three times.

#### 2.7.3 Assay for cell membrane integrity and SEM observation

Antibacterial activity was further investigated according to Agrawal et al. [13] for their possible effects on targeted cells by scanning electron microscopic analysis. Test organism was grown overnight to mid-logarithmic phase or at a fixed OD of 1.5 at 600 nm. The organism in the present study was treated with 1× MIC concentration of organic active fraction and incubated at 37 °C with continuous shaking. After 18 h the culture was centrifuged at 5,000 rpm for 5 min; the supernatant was removed and washed three times with autoclaved water. Test organisms were then fixed in 2.5% glutraldehyde in 25 mm phosphate buffer solution (PBS) for 5–6 hours at 4 °C, then washed with PBS 3 times for 10 min. Then the sample was dehydrated with an ethanol series (30%, 50%, 70%, 90% and 100% ethanol). Samples were dried using a critical point dryer (Emitech K850, Berkshire, U.K.) followed by sputter coating with gold and palladium at 120 mA for 15 mins using sputter coater (Quorum Technologies SC7620, Berkshire, U.K). The samples were scanned under SEM (Model EVMA10, ZEISS, Germany) at TERI-Deakin Nano Biotechnology Centre, New Delhi, INDIA.

### 2.8 Antioxidant capacity

#### 2.8.1 DPPH-radical scavenging activity

Antioxidant activity of the ethyl acetate extract was determined by DPPH free radical scavenging assay as described by Clarke et al. [22] with slight modification. 20 μL of extract in water was mixed with 180 μL of DPPH (obtained from Sigma) in methanol (0.1 mM) in a 96-well plate. The plate was kept in the dark for 30 min, after which the absorbance of the solution was measured at 517 nm in a microtiter plate-reader (iMark^™^ Microplate Reader, Bio-Rad). Appropriate blank (methanol) and standard (ascorbic acid solutions in water) were run simultaneously. Ethyl acetate extract was first screened at a single concentration of 1 mg/mL. The data are expressed as mean percentages and the results were repeated in triplicate. Inhibition of the DPPH free radical in percentage (I%) was calculated as:

$$\text{I}\%=\left[\left({\text{A}}_{\text{control}}-{\text{A}}_{\text{sample}}\right)/{\text{A}}_{\text{control}}\right]\times 100$$

Where A _control_ = absorbance of the control reaction without the tested extract and A _sample_ = absorbance in the presence of the sample.

Decreased absorbance of the reaction mixture indicates stronger DPPH free radical-scavenging activity.

#### 2.8.2 ABTS radical scavenging activity

The ABTS^+^ scavenging capacity was determined using the method described by Long and Halliwell et al., [23] with minor modification. Ammonium persulfate was added (2.45 mM final concentration) to the stock solution of ABTS (7 mM) prepared in water and the solutions were allowed to react for 24 h in dark. To obtain an absorbance of 0.70 at 734 nm the solutions was diluted using methanol solution. Afterward 10 μL of test sample, 200 μL ABTS (obtained from Sigma) was added to wells. Only 210 μL of ABTS solution was used as negative control (A0). The reaction was left to proceed in the dark at room temperature for 20 min before the measurement of absorbance at 734 nm with a microplate reader. Gallic acid was used as a positive control. Ethyl acetate extract was first screened at a single concentration of 1 mg/mL. The percentage ABTS scavenging activity was indicated by the reduction in the absorbance of ABTS radical and was computed using the formula expressed below:

$$\%\text{ABTS}\;\text{scavenging}\;\text{activity}=\left[\left(\text{A}0-\text{A}1\right)/\text{A}0\right]\times 100$$

### 2.9 Total phenolic content determination with Folin–Ciocalteu’s reagent method

**Fig 1:**
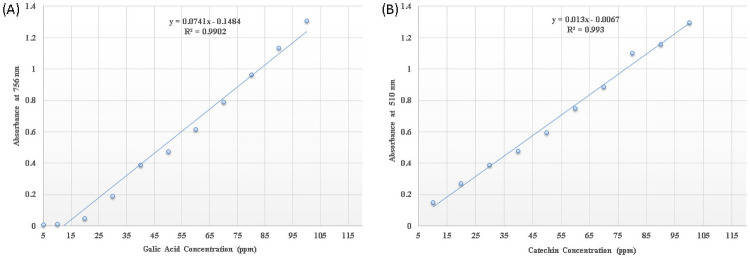
Representative standard curves for (A) total phenolic content; (B) flavonoid content.

The total phenolic of the ethyl acetate extract was determined by Folin-Ciocalteu method as described by Singleton et al. [24], using gallic acid as the phenolic standard. Extract in the concentration of 1 mg/mL was used in the analysis. Briefly, 100 μL of sample was mixed with 500 μL of 10% Folin-Ciocalteu reagent and incubated for 5 min at room temperature. Then 500 μL of 7.5% aqueous sodium bicarbonate (NaHCO_3_) was added to solution and was mixed thoroughly. The solution was incubated at 37 ºC in dark for 2 hours. Blank was concomitantly prepared, containing 100 μL methanol, 500 μL 10% Folin-Ciocalteu’s reagent and 500 μL of 7.5% of NaHCO_3_. The absorbance was observed spectrophotometrically at λmax = 765 nm for triplicate sample and the mean value of absorbance was achieved. The standard calibration curve was construed by using gallic acid as a standard (Fig 1A). The concentration of phenolics were estimated (mg/mL) from the calibration line, the content of phenolics in the extract was expressed in terms of gallic acid equivalent (mg of GAE /g of extract).

### 2.10 Flavonoid content determination based on aluminum–flavonoid complexes formation method

The total flavonoid estimation was done by method described by Herald et al. [25] using the spectrophotometric 96 well-microplate with minor modification. Firstly, 100 μL of distilled water was added into each of the 96 wells, followed by 10 μL of 50 g/L NaNO_2_ and 25 μL of standard or ethyl acetate extract. After 5 min, 15 μL of 100 g/L AlCl_3_ was added to the mixture and incubated for 6 min at room temperature. The plates were further added with 50 μL of 1 M NaOH and 50 μL of distilled water and absorbance was measured at 510 nm. The calibration curve (average R2 = 0.9930) was generated using catechin at different concentrations ranging from 5–250 μg/mL (Fig 1B). Then the flavonoid content in the extract was determined from the graph and expressed in terms of catechin equivalent (mg of CAE/g of extract).

### 2.11 TLC-bioautography assay

A thin layer chromatography combined with bioautography was conducted with slight modifications from the methods described by Feng Pan et al. [26]. The ethyl acetate extract was subjected to TLC on four TLC plates, silica gel (Merck Millipore, India). The plate was developed in chloroform: methanol (8:2). Chromatographic were observed using ultraviolet lamps emitting at 365 nm, followed by heating for 3 min at 105 °C after being sprayed with vanillin sulphuric acid (for phenol and terpenoids). The second plate was visualized at 365 nm, followed by spraying with ninhydrin solution (for amino acid and peptide). The third plate was sprayed with anisaldehyde-sulphuric acid reagent for detection of flavonoid, terpenes, and saponins. The fourth TLC plate was sprayed with 0.04 mg/mL DPPH in ethanol for TLC based bioautography detection for antioxidants, and the presence of yellow stains was indicative of components with antioxidant activity.

### 2.12 Gas Chromatography–Mass Spectrometry (GC–MS) analysis

Gas chromatography-mass spectrometry (GC–MS) analysis was conducted based on previously developed protocol by Panigrahi et al. [27] with slight modifications. The Agilent Technologies 6980N (GC) equipped with 5979 Mass Selective Detector (MS), HP-5MS (5% phenyl methyl siloxane) capillary column of dimensions 60.0 m × 250 μm × 0.25 μm and helium as carrier gas at 1 mL/min were used for the analysis. The column temperature was maintained initially at 50 °C for 5 min, followed by an increase of 10 °C/min to 250 °C and was kept isothermal for 5 min. The MS was operating at 70 eV. The constituents were identified by comparison of their mass spectral data with those from W9N11 Spectral Library.

## 3. Results

### 3.1 Isolation and identification of fungi by ITS sequencing

**Fig 2:**
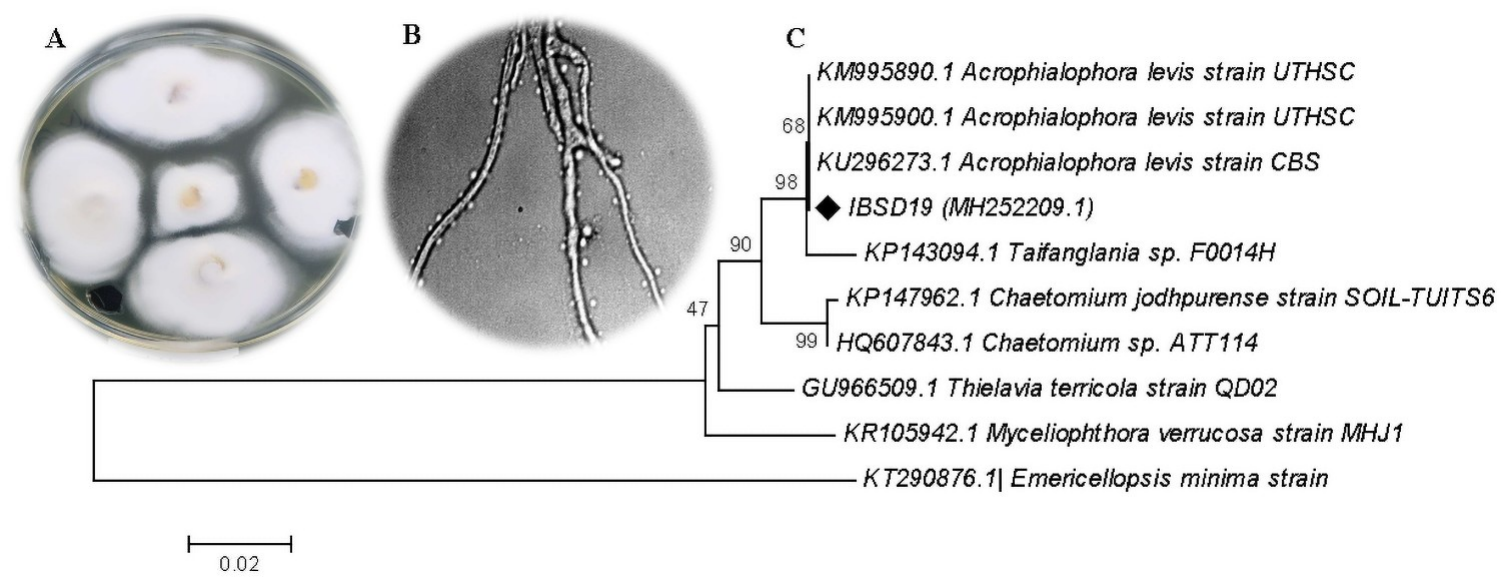
(A) Colonies of culture IBSD-19 grown on PDA for seven days, (B) image of conidia and conidiophores from a seven-day MEA culture, (C) phylogenetic tree of partial ITS-rDNA sequences of IBSD19 fungal strain. Reference sequences were downloaded from NCBI with the accession numbers indicated in parentheses.

The thermophilous fungus IBSD-19 was isolated from the sediment samples collected from the hot water spring at Meghalaya India where temperature was recorded 50 ºC during sample collection. The fungus was identified using morphological and molecular characterization. Based on BLAST search analysis of the sequence, the isolate showed maximum identity with *Acrophialophora levis* (99%) having accession number KM995890.1 (Fig 2a–2c). The sequence of the isolate *A*. *levis* IBSD-19 has been deposited in GenBank with accession number MH252209.1. The sample culture was deposited in the microbial repository center of Institute of Bioresources and Sustainable Development, Imphal, India with reference number MRC 8226.

### 3.2 Anti *Staphylococcus aureus* and anti MRSA activity

**Fig 3:**
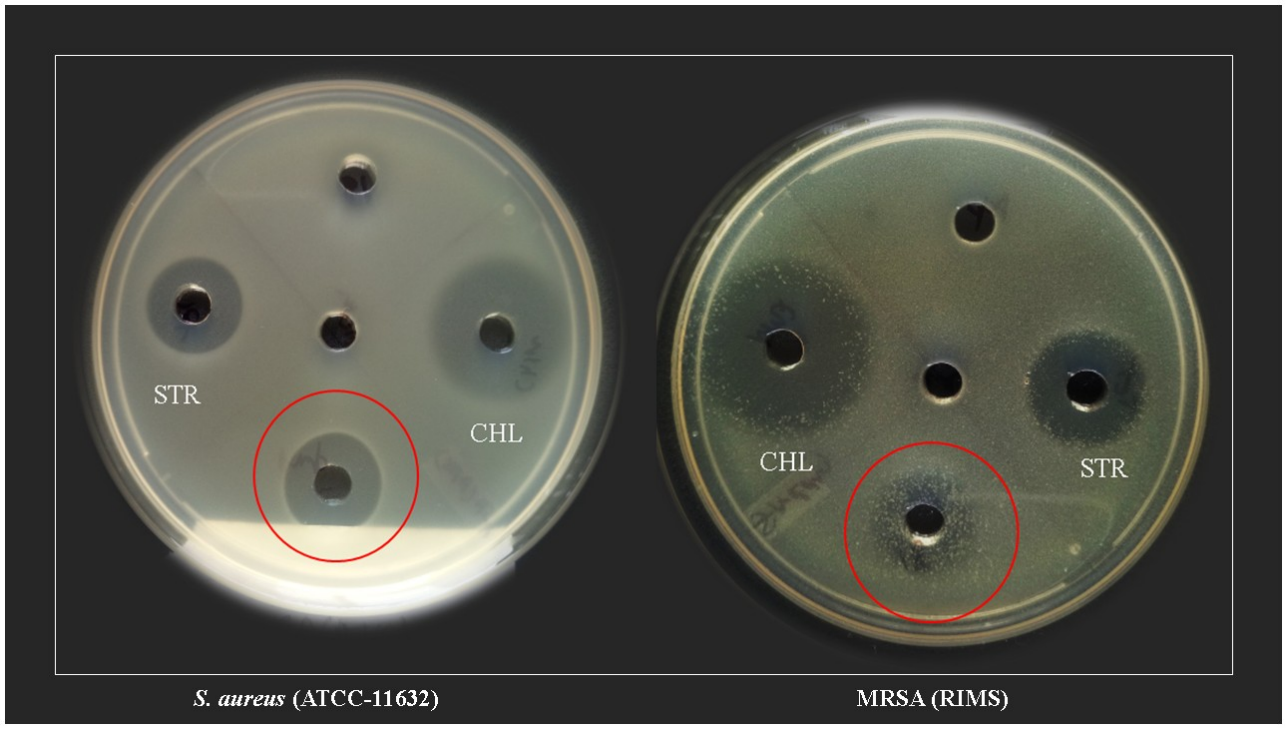
Antibacterial potential of ethyl acetate extract against *S*. *aureus* and MRSA (STR = streptomycin; CHL = chloramphenicol).

The antibacterial activity of all three organic fractions of *A*. *levis* IBSD-19 was assessed using the agar well diffusion method by measuring the diameter of growth inhibition zones at a fixed concentration of 1 mg/mL. We found that both *S*. *aureus* and MRSA were only sensitive to ethyl acetate fraction with the inhibition zone of 13.2 and 10.1 mm (Fig 3a and 3b) respectively. However, MRSA have less sensitivity than other strain to ethyl acetate fraction. The minimal inhibitory concentrations of the ethyl acetate extract were 1 μg/mL for *S*. *aureus* and 4 μg/mL for MRSA.

### 3.3 Mechanism of action of fungal metabolites against *S*. *aureus* and MRSA

**Fig 4:**
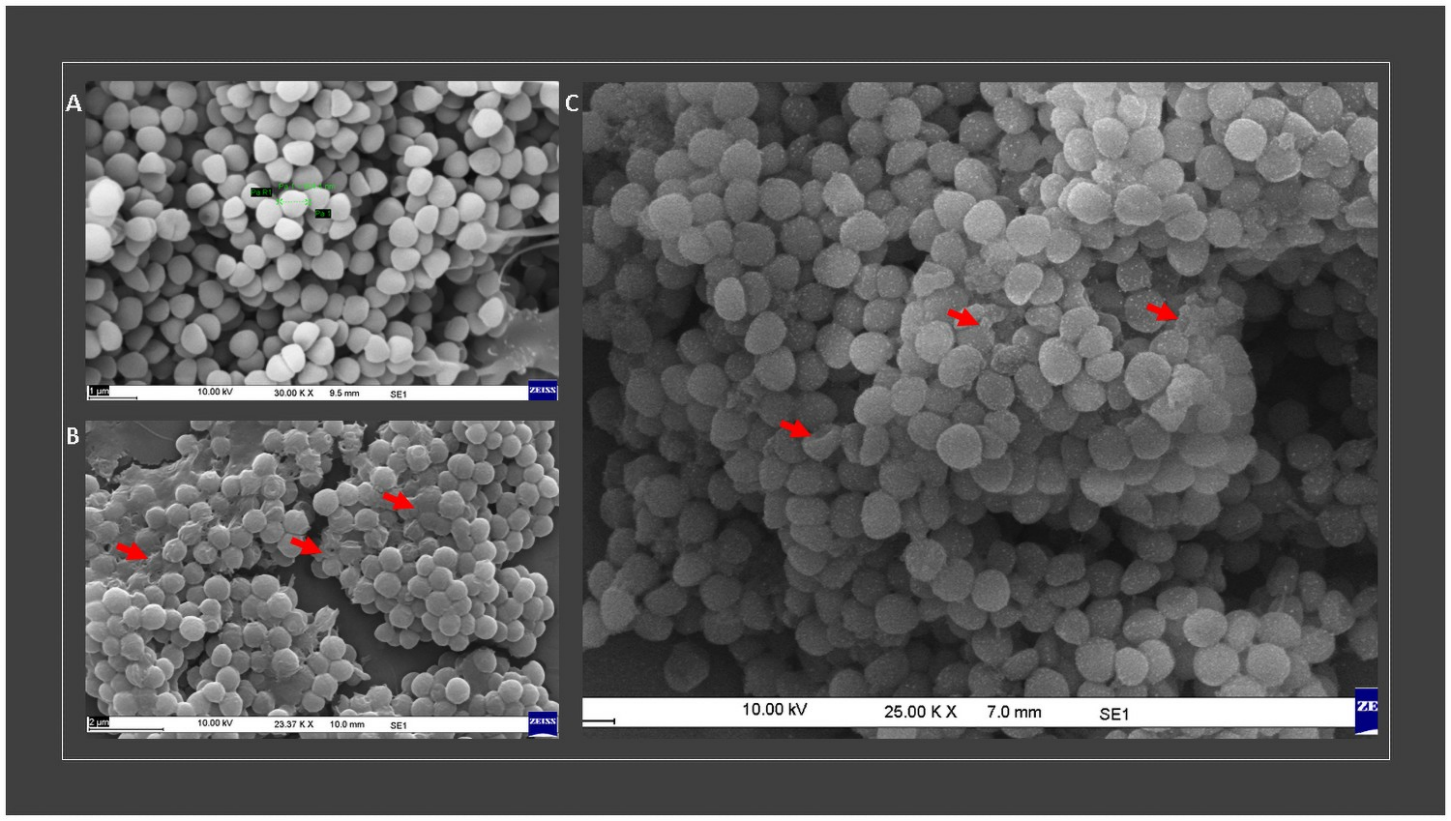
Effects of ethyl acetate extract on the ultrastructure of *S*. *aureus* and MRSA as observed by SEM (A, control; B, *S*. *aureus*; C, MRSA).

For the SEM analysis, both *S*. *aureus* (ATCC 11632) and clinical MRSA isolates were selected for the treatment as this clinical isolate resistant to oxacillin, cefoxitin and amoxicillin, was found to be susceptible to the ethyl acetate extract (MIC = 4 μg/mL). Regular morphology was seen in *S*. *aureus* not treated with extract, with uniform cells in size and distribution, as well as a flat surface (Fig 4a). On the other hand, cell membrane rupture was observed with surface depressions, and biconcave appearances, in cells in contact with extract (Fig 4b and 4c).

The formation of holes on the cell surface and leakages of intracellular components were visible when the cells were incubated for 18 h with ethyl acetate fraction at the 1×MIC (2 μg/mL for *S*. *aureus* ATCC 11632 and 8 μg/mL for MRSA). The majority of ethyl acetate fraction treated cells in many fields exhibited central depression or collapses with discernible holes on the cell surfaces.

Irregular cluster formation of deformed *S*. *aureus* ATCC 11632 cells was observed when exposed to 1× MIC. This observation was consistent with the previous observation made by Greenwood and O’Grady [28]. These observations taken together indicate that ethyl acetate fraction may contain unique antibacterial molecules exerting altered cell metabolism and impaired cell replication through membrane-targeted pore formation and membrane disruption, which rarely induce drug-resistance in bacteria [29]. This study suggests that since the test fraction had strong anti-staphylococcal and anti-MRSA activity by probably acting on the cell surface, it could present a low risk of developing drug resistance, and therefore this fraction might be thought as an alternative to traditional antibiotics, especially those used for the treatment of skin and mucosal infections.

### 3.4 Antioxidant activity determination

The antioxidant activity of the thermophilous fungi IBSD-19 was evaluated using several methods, viz. ABTS assay and the DPPH radical scavenging assay.

#### 3.4.1 DPPH radical scavenging activity

**Fig 5:**
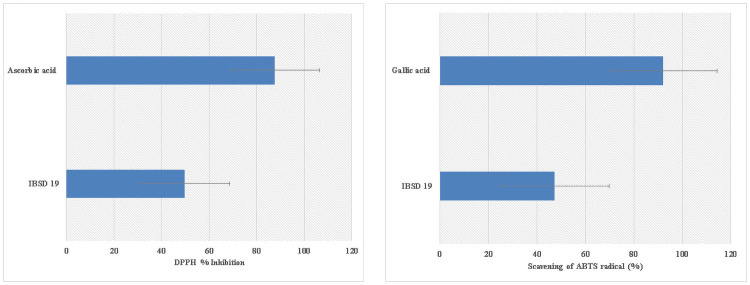
(A) DPPH radical scavenging activity of the extract. (B) ABTS+ radical scavenging activity of the extract. Values are mean ± SD; n = 3.

DPPH is a simple and robust antioxidant activity screening assay. The DPPH assay is used to assess the free radical scavenging ability of a substance/compound by using a stable free DPPH radical. A substance/compound which can transfer hydrogen atoms or electron to the DPPH delocalization of the spare electron over the DPPH molecule that gives rise to its deep violet color [30]. This study demonstrated that ethyl acetate extract exhibited DPPH radical scavenging activity based on the color changes observed from the violet DPPH radical solution into yellow-colored diphenyl picrylhydrazine (reduced form). The result showed that the extract showed significant DPPH radical scavenging activity. At a concentration of 1 mg/ml the extract scavenged 50% DPPH radicals (Fig 5A). This is suggesting that the ethyl acetate extract exhibits hydrogen donating ability.

#### 3.4.2 ABTS+ radical scavenging assay

The ABTS assay was another antioxidant assay used to evaluate the radical scavenging activity of thermophilic fungal ethyl acetate extract. This assay involves the use of ABTS.+, a stable radical cation that can be generated chemically by reacting ABTS with potassium persulfate. Thermodynamically, the ABTS. + can be reduced by compounds which have a lower redox potential than that of ABTS(0.68 V) [31]. This assay revealed that the addition of fungal extract decolorized the intensely blue green ABTS. + solution in, suggesting that the ethyl acetate extract is capable of reducing the blue-green color of ABTS. + back into ABTS, which is colorless. The results showed that IBSD-19 ethyl acetate extract exhibited significant ABTS radical scavenging activity (p < 0.05) 47% at a concentration of 1 mg/mL (Fig 5B).

### 3.5 Phenolic and flavonoid contents of IBSD-19 extract

The TPC of ethyl acetate extract was estimated using the Folin–Ciocalteu’s reagent method. The Folin–Ciocalteu’s reagent method is based on the measurement of the total concentration of phenolic hydroxyl group which reacts with Folin–Ciocalteu’s reagent to form blue complexes in the extract. The result showed the development of blue color complex with extract as measured by the absorbance at 750 nm. This is suggesting the presence of phenolic compounds in the extract. Phenolic compounds may contribute directly to antioxidative action. The total phenolic content was 50 mg GAE/g dry weight of extract. Meanwhile, the flavonoid content determination assay showed lower concentration of flavonoids in the IBSD-19 ethyl acetate extract as compare to phenolic. The total flavonoid content was measured 20 mg CAE/g of dry weight.

### 3.6 Combined TLC-bioautography analysis

**Fig 6:**
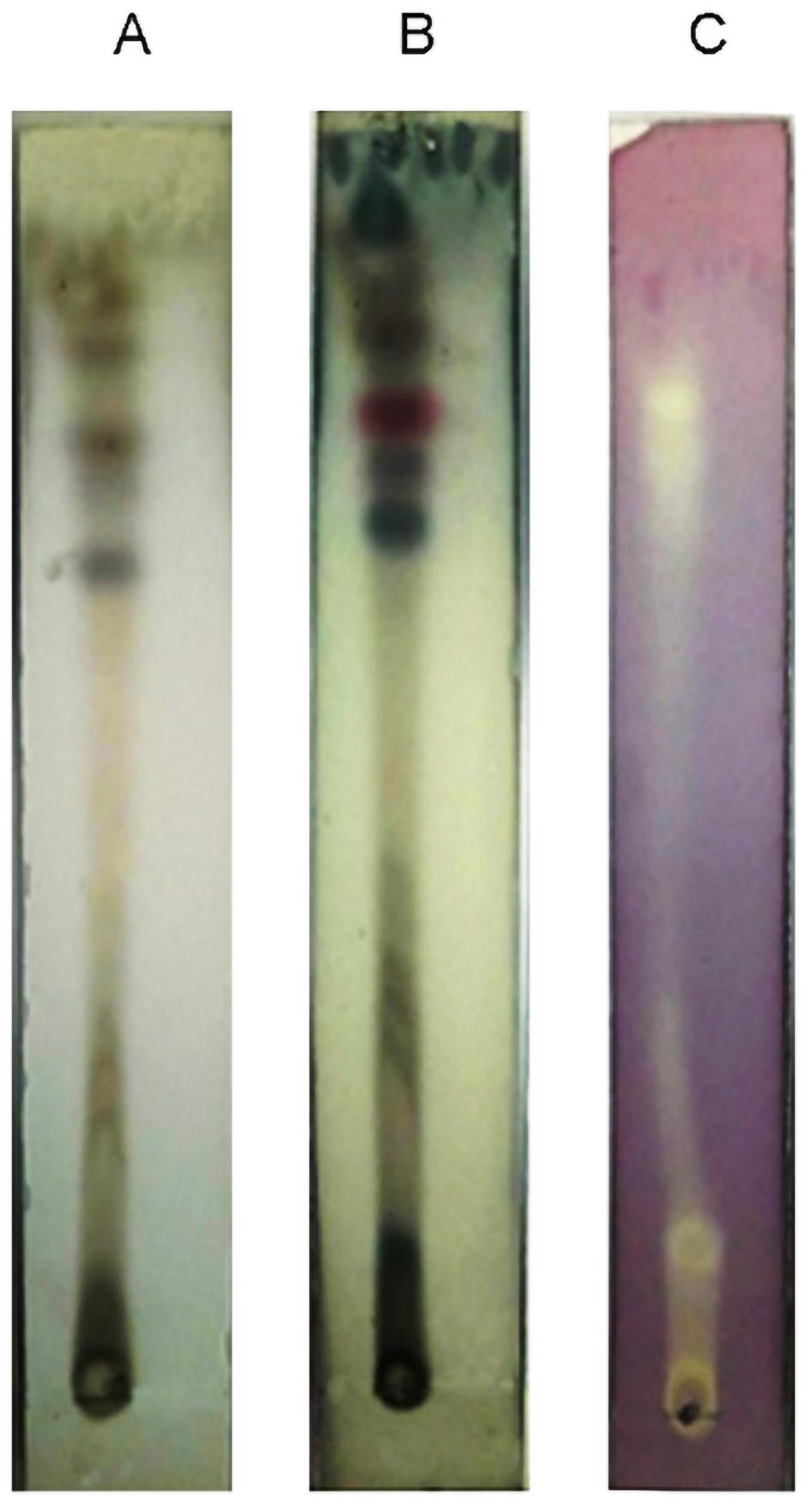
TLC photography of fungal extract. These fractions were developed in a preselected solvent system containing chloroform /methanol (8:2, by volume) and visualized using several methods, namely, ultraviolet lamps emitting at 365 nm after heating for 3 min at 105 °C after spraying with vanillin sulphuric acid (A), ultraviolet lamps emitting at 365 nm after spraying with anisaldehyde-sulphuric acid solution (B), and 0.04 mg·mL−1 DPPH in ethanol (C).

Separation of the compound using thin layer chromatographic (TLC) is a rapid and sensitive method for the detection of bioactive compounds. Ethyl acetate extract was subjected to combined TLC-bioautography to detect the diversity of the metabolic composition and antioxidant metabolites. Out of the various mobile phases tested, chloroform: methanol (8:2, by volume) gave the best resolution of compounds (Rf = 0.05–0.95). Further heating of the plate for 3 min at 105 °C the plates were sprayed with vanillin sulphuric acid and multiple spots have been appeared as shown in (Fig 6A). In addition, when visualized using p-anisaldehyde–sulfuric acid solution the appearance of purple or dark green spots may indicates the presence of flavonoid and saponin respectively in the extract (Fig 6B). For bioautography assay, TLC plate sprayed with DPPH solution (Fig 6C) exhibited three spots showing good antioxidant activity. The baseline of TLC plate also showed deep yellow color formation, which also suggesting the presence of antioxidant constituents. The result indicates that this thermophilic fungus produces various metabolites containing multiple flavonoid compounds, saponin and phenols, which could remove DPPH free radicals.

### 3.7 Chemical profiling of thermophilous fungus IBSD-19 ethyl acetate extract using GC–MS analysis

**Fig 7:**
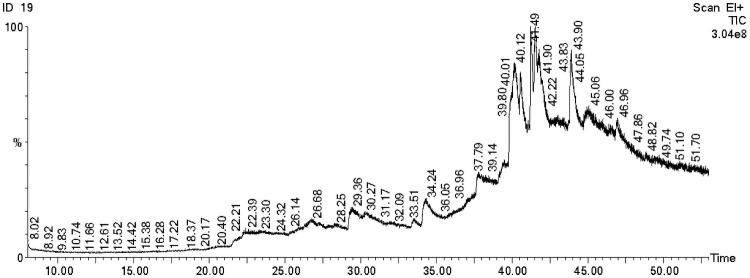
GC-MS based chemical profiling of ethyl acetate extract from IBSD-19 fungi strain.

To determine the partial chemical constituents that may be responsible for biological activity, IBSD- 19 ethyl acetate fraction was subjected to GC/MS analysis. In the present study, GC/MS analysis successfully detected esters, alcohols, phenols and cyclic dipeptides in the complex mixtures of ethyl acetate extract. These chemical compounds were identified by comparison of their mass spectra to the database available on the W9N11 MS library. The detailed information of the chemical compounds based on their retention time, molecular weight and molecular formula are listed in Table 1 and their chemical structures are depicted in **Figs** 7 **and** 8.

**Fig 8:**
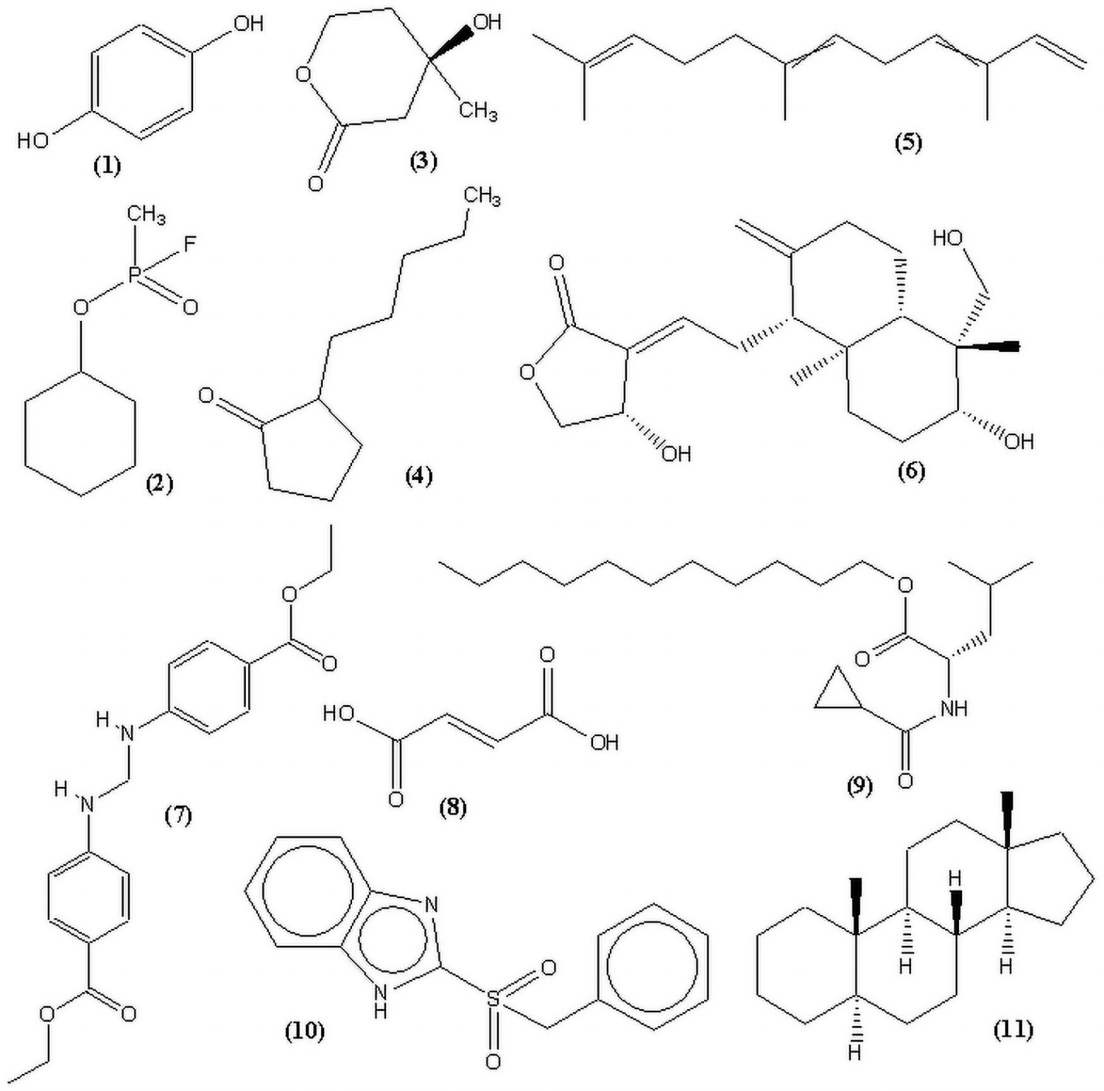
Chemical structures of constituents detected in IBSD-19 extract.

**Table 1:** List of major compounds identified from IBSD-19 fungal extract.

No.	Retention Time (min)	Compound	Molecular weight	Molecular formula	Peak area (%)	Reported activity	Reference
1.	22.39	Hydroquinone	110	C_6_H_6_O_2_	0.179	Antioxidant	[48]
2.	26.68	Cyclosarin	180	C_7_H_14_	0.510	Acetylcholinesterase inhibiter	[49]
3.	29.45	Mevalonolactone	130	C_6_H_10_O	0.787	Antibacterial	[50–52]
4.	34.32	2-Pentylcyclopentanone	154	C_10_H_18_	1.292	Antioxidant	[53]
5.	37.79	α- Farnesene	204	C_15_H_24_	2.305	Antibacterial and antioxidant	[54–56]
6.	39.53	Andrographolide			0.891	Antibacterial	[57–59]
7.	40.16	Bis (4-ethoxycarbonylphenylamino) methane	342	C_19_H_22_	11.148	-	[60]
8.	41.55	Fumaric acid derivative	352	C_21_H_36_O_4_	3.506	Antibacterial	[61, 62]
9.	41.77	L-Leucine, N-cyclopropyl carbonyl-, undecyl ester	353	C_21_H_39_NO_3_	17.170	-	-
10.	43.90	Benzimidazole,2- (benzyl sulfonyl)	272	C_14_H_12_N_2_O_2_S	8.746	Antifungal	[63, 64]
11.	45.06	Androstane derivative	481	C_29_H_43_	5.328	Antibacterial	[65]

## 4. Discussion

The secondary metabolites of the living organisms in extreme environments which have extreme characteristics are very important sources having potential to discover novel pharmacological agents including antimicrobial and anticancer agents. Thermophilic fungi that develop various adaptation mechanisms to adapt to this type of habitats are remarkable as new natural metabolite sources. Temperature is one of the most important parameters that affect the growth and survival of microorganisms. Thermophilic organisms that can tolerate high temperatures is narrower in Eukarya than in prokaryotes and species that tolerate temperatures above 61°C are very rare [32]. However, thermophilous fungi are those which show temperature optima in the range of 25 to 55°C [33]. The occurrence of thermophilous fungi is now known to be ubiquitous. In India these fungi have also been reported from various substrates [33]. Among the eukaryotes, certain fungi have the capability to remain active and proliferate at high temperatures. It is believed that to survive in such extreme environment, these fungi have developed unique metabolic systems that have often leads to produce novel bioactive molecules. For example, with an increase in temperature, there is an increase in the proportion of saturated fatty acids incorporated into phospholipids, whereas at lower temperature, a higher proportion of unsaturated fatty acids is incorporated. This phenomenon is called homeoviscous adaptation [34]. It was observed that in fungus *T*. *lanuginosus* the concentration of linoleic acid (18:2) was twofold higher at 30°C than at 50°C. The degree of unsaturation of phospholipid fatty acids was 0.88 in mycelia grown at 50°C but 1.0 in the temperature-shifted cultures (from 50 to 30°C) and 1.06 in cultures grown at constant 30°C [35]. A decrease in the degree of unsaturation was also observed in *Chaetomium thermophile* when it was subjected to heat shock [36]. Several researchers have reported thermophilic fungi from diverse environmental habitats such as geothermal sites and hot springs [32]. In this study, an antibacterial and antioxidant producer fungus was isolated, from the Bakra natural hot spring in India. To our knowledge, this report is the first on *Acrophialophora levis* fungi isolated from the hot spring environment. Previously *Acrophialophora levis* a thermophilic and thermotolerant fungi was isolated from Iraqi soils which can grow at 45°C and was found to be a significant producer of industrial enzymes [37]. Likewise, in previous studies *Acrophialophora* sp. was extensively studied for production of industrial enzyme [38–43] however, no report was present on antibacterial and antioxidant activity. This study reports for the first time, the potential source of anti- *S*. *aureus* activity from a thermophilous fungi *Acrophialophora levis* isolated from Bakra natural hot spring. Phytochemical analysis of ethyl acetate extract confirms the presence of alkaloids, phenols, flavonoids, saponins, and terpenes. Previous studies conferred antibacterial activity of phenol and phenolic acids against *Escherichia coli*, *Pseudomonas aeruginosa*, *S*. *aureus*, and *Listeria monocytogenes* [44] found that gallic acid causes irreversible alterations in cell membrane properties, such as alterations in hydrophobicity, a decrease in the negative surface charge and local rupture or formation of pores in the cellular membranes, with consequent releasing of intracellular constituents, as occurred in the present study. The mechanism of antibacterial action of phenolic compounds is not fully understood; however, bacterial cell membrane rupture is one of the main mechanisms reported in both Gram-positive and Gram-negative bacteria [45], which reinforces the hypothesis that the anti *S*. *aureus* action found in fungal extract is largely due to the presence of such types of compounds. Similarly, the correlation between total phenolic content and antioxidant potential of any sample had already reported in previous studies [46]. The existence of hydroxyl groups indicates the free radical scavenging ability of phenols. Because of the high total amount of phenolic content, the antioxidant activity of the extract was high. The strong correlation between the two assays measuring antioxidant capacity and the TPC suggests that phenolic compounds make a large contribution to the antioxidant properties of IBSD-19 extract. Literature has shown that TPC had strongly associated with DPPH and ABTS+ antioxidant capacities [47]. However, further characterization of the extract is required to verify the exact molecule associated with the antibacterial and antioxidant capability of IBSD-19 extract. According to these findings, thermophilous fungi are potential source of secondary metabolites, which can be beneficial in infectious and oxidative stress related diseases. Today materials of natural origin have become preferable in drug investigations because of their lower side effects and minimum toxicity on human health. Therefore, the *Acrophialophora levis* strain IBSD-19 is a promising fungus for the biotechnological production of antibiotics and antioxidant substances. This study highlights the need for more research in extreme environments.
